# Digital Health Interventions in Everyday Settings

**DOI:** 10.3390/ijerph17082702

**Published:** 2020-04-15

**Authors:** Katrien De Cocker

**Affiliations:** Institute for Resilient Regions, University of Southern Queensland, Springfield Central QLD 4300, Australia; katrien.decocker@usq.edu.au

Digitalisation, the use of digital technologies and platforms such as computers, websites, smartphones, and wearable devices, is everywhere in many ways [1]. Through digital, electronic, mobile, and machine-learning innovations, many organisations and businesses, including the health sector, have changed and improved their daily processes and systems in recent decades [2]. For the health sector, this so called ‘Fourth Industrial Revolution’ resulted, for example, in opportunities to ease complex health-related data and information recording and analysing (health informatics), improve clinical diseases diagnosis (health care), and allow self-care and self-monitoring in patients with chronic diseases (health education). Furthermore, in health promotion practice, we see the influence of digital, electronic (eHealth) and mobile (mHealth) technologies (e.g., devices, apps, gamification, ‘smart objects’), aiming to improve physical and mental health behaviours and outcomes [3]. Nowadays, the sky seems the limit when it comes to designing new digital health interventions. The question remains, however, whether and how these innovations affect health promotion interventions in everyday settings, such as primary care, communities, workplaces, and schools, and how lay people, including older-aged users, respond to them [4,5]. One of the challenges of digital health interventions, for example, is the improvement of low initial and continued user engagement. This Special Issue of the International Journal of Environmental Research and Public Health brought together some of the latest research on novel digital approaches used in health promotion interventions in various settings of daily life.

In this Special Issue, we present four original studies (mainly based on experimental trials) conducted in various countries throughout the world (Germany, Bangladesh, USA., Hong Kong) and one systematic review with a meta-analysis of RCTs and quasi-experiments, completed by Australian researchers. All contributions span adult populations of various profiles, including fragile older adults, chronic disease patients, students, and adults at risk of cognitive impairment. The main settings in which the studies were conducted were primary care, schools, and fitness facilities. Outcomes were diverse, comprising data accuracy, patient communication and treatment adherence, willingness to use smart objects to improve medication adherence, engagement with a health promotion web-platform, and cognitive impairment.

Hasan and colleagues designed and developed a software system to detect and reduce healthcare data errors, by analysing records of anthropometric data of over 40,000 patients. This study shows the potential to use digital health in lower income countries. The results should help to design a portable health clinic box to use in remote healthcare systems of developing countries where infrastructure is limited. In the systematic review of Yadav and colleagues, it was examined how digital communication interventions (compared to providing usual health information) can improve osteoporosis treatment adherence and prevent secondary fractures in patients aged 50 and above recovering from fragility fractures. The meta-analysis shows that digital interventions were twice as effective. The study of Choi provides evidence regarding the emerging availability of smart objects in health interventions. In this study, the acceptability of a smart pillbox was tested among patients aged 40 and over with chronic diseases. About 71% of participants were willing to use a smart pillbox for free. The willingness to use this smart pillbox was larger in those with heart disease, those who had missed taking a medication dose in the past, those with a higher daily medication intake, and those with a higher income. Older patients were less willing to use the smart pillbox. Further, the study of Stassen et al. confirms that low engagement remains a weakness of digital health interventions. In an 8-week web-based intervention promoting health behaviours (with vs. without face-to-face contact) among vocational school students, website log-ins were low (16.6%). The likelihood of initial engagement was, however, higher for those having additional face-to-face contact, for female users, and for those with the skills to deal with health information. Finally, the study of Palace and colleagues shows the potential of digital health interventions to not only target physical health outcomes and behaviours, but also cognition-related issues. The authors pilot-tested an iPad-enhanced aerobic exercise intervention delivering virtual 3D tours in picturesque landscapes (vs. aerobic exercise only). This digital health intervention was designed to enhance wayfinding outcomes and cognitive functioning among adults at risk for cognitive impairment. The virtual reality-enhanced aerobic exercise may be an effective method for improving cognitive function and increasing confidence to navigate in day-to-day situations among individuals at risk of cognitive impairment.

The practical implications learned from this Special Issue include that co-created, person-centred, digital approaches to monitor and educate older people with fragility fractures are promising. In addition, potential strategies to increase intervention engagement among student risk subgroups contain the provision of some form of face-to-face contact, fostering health-related skills and the option to use a web-based platform in everyday teaching. Furthermore, the use of smart objects and tablet-enhanced aerobic exercise intervention appears feasible and acceptable for middle-aged chronic disease patients and adults at risk of cognitive impairment respectively.

In summary, this collection of studies confirms the endless options in terms of using digital innovations in everyday settings for adults of various age groups and with different backgrounds. However, the present results might also indicate that the full potential of digital health interventions in everyday settings has not yet been reached and certain challenges remain in place. As such, there is still more research to be conducted; for example, regarding long-term established user engagement in digital interventions, the optimal integration of diverse (digital) resources addressing multiple health issues and behaviours, and the (cost-) effectiveness of smart objects and willingness to pay for them.
